# Psychopathic traits among a consecutive sample of Finnish pretrial fire-setting offenders

**DOI:** 10.1186/s12888-015-0425-x

**Published:** 2015-03-10

**Authors:** Annika Thomson, Jari Tiihonen, Jouko Miettunen, Eila Sailas, Matti Virkkunen, Nina Lindberg

**Affiliations:** Kellokoski Hospital, Kellokoski, 04500 Finland; Niuvanniemi Hospital, Kuopio, 70240 Finland; Department of Psychiatry, University of Eastern Finland, Kuopio, Finland; Department of Clinical Neuroscience, Karolinska Institutet, Stockholm, Sweden; Center for Clinical Neurosciences, Department of Psychiatry, University of Oulu and Oulu University Hospital, Oulu, Finland; Medical Research Center Oulu, University of Oulu and Oulu University Hospital, Oulu, Finland; Center for Life-Course and Systems Epidemiology, University of Oulu, Oulu, Finland; Forensic Psychiatry, University of Helsinki and Helsinki University Hospital, Helsinki, Finland

**Keywords:** Arson, Fire-setting behavior, Firesetter, PCL-R, Psychopathy

## Abstract

**Background:**

Psychopathy, a severe disorder of personality, is well represented in the criminal and forensic psychiatric population and is significantly associated with increased risk of violence and crime. Fire-setting is a major source of property damage, injury, and death in many Western countries. The primary aim of this study was to evaluate psychopathic traits in a consecutive sample of Finnish male pretrial fire-setting offenders. Further, we wanted to investigate whether fire-setting recidivists show higher traits of psychopathy than one-time firesetters and whether exclusive firesetters show lower traits of psychopathy than those with criminal versatility.

**Methods:**

The forensic psychiatric examination statements for male firesetters who underwent a pretrial forensic psychiatric evaluation during a 10-year period (1989 –1998) were reviewed. The sample comprised 129 firesetters with normal IQ, 41 of whom were fire-setting recidivists. Fifty men were exclusive firesetters. Assessment of psychopathy-like personality character was performed using the 20-item Hare Psychopathy Checklist-Revised.

**Results:**

Two individuals (1.6%, 95% Cl: 0.0-3.7) scored ≥30 points and 19 (14.7%, 95% Cl: 8.6-20.8) ≥ 25 points on the PCL-R. The mean PCL-R total score was 16.1 (SD 6.88), the mean Factor 1 score 5.0 (SD 3.41), and the mean Factor 2 score 9.9 (SD 3.86). No significant differences emerged between the recidivists and the one-time firesetters. The versatile firesetters exhibited significantly higher mean total and factor scores than the exclusive ones.

**Conclusion:**

Among firesetters, there is a subgroup of persons with significant psychopathic traits, which should be recognized in legal and health care organizations. Although psychopathy was associated with greater criminal versatility, it bore no relationship to fire-setting recidivism.

## Background

Psychopathy, a severe disorder of personality, is defined as a constellation of affective, interpersonal, and behavioral characteristics, including impulsivity, irresponsibility, shallow emotions, lack of empathy, guilt, or remorse, pathological lying, and persistent violation of social norms and expectations [1-3]. At the interpersonal level, psychopathic individuals have been described as grandiose, arrogant, callous, dominant, superficial, and manipulative. Affectively, they are short-tempered and unable to form strong emotional bonds with others. These interpersonal and affective features are associated with a socially deviant lifestyle that includes irresponsible behavior and a tendency to ignore or violate social conventions and morals [2]. The prevalence of psychopathy is less than 1% in general populations, but it is highly prevalent among prison populations [4]. Offenders with psychopathy typically begin their antisocial and criminal activities at a relatively young age and continue to engage in these activities throughout their lifespan [5]. In addition, their use of violence tends to be more instrumental, dispassionate, and predatory than that of other offenders [6]. A psychopathic character is related to poorer treatment compliance and a higher dropout rate [7,8]. Psychopathic criminals re-offend more quickly and more often following release from custody than do other offenders [9]. All in all, both legal and medical authorities should identify this high-risk group of offenders characterized by versatile and repeated offending behavior and modest treatment results.

Fire-setting is a major source of property damage, injury, and death in many Western countries [10,11]. While pyromania is a rare psychiatric disorder even among firesetters [12], other psychiatric disorders, including antisocial and other personality disorders, schizophrenia, mental retardation and organic psychosis as well as mood disorders, appear to be prevalent [13,14]. Some researchers consider firesetters as a dangerous group of offenders who are highly likely to repeat this behavior [15]. However, recently, the opposite has also been reported [16]. Besides excitement and delusions, also anger, revenge, vandalism and attention-seeking have been described as motives for fire-setting behavior [17,18]. Among a sample of 138 randomly selected cases of persons arrested for arson in New York City, more than half of the cases were persons who used fire as a weapon to gain revenge. Insurance fraud, welfare fraud, and crime concealment accounted for approximately one-fifth of the arson arrests [19]. According to Harris and Rice [20] and Lambie et al. [21], fire-setting recidivists show high levels of aggression as well as high rates of recidivism for all crime types. In fact, in a study by Ducat et al. [16], the vast majority of fire-setting recidivists were criminally versatile. Firesetters with criminal versatility are more likely to be personality disordered than those who commit only fire-setting crimes [22]. On the other hand, firesetters have also been described as more shy, withdrawn, and socially isolated and less likely to be physically aggressive than other mentally disordered patients sent to a maximum security psychiatric facility [17]. It is reported that they show poor assertiveness and communication skills and low self-esteem [23] as well as exhibit more suicidal and self-destructive behavior than other criminal offenders [24]. Thus, firesetters show marked heterogeneity in their personality and motives.

Identification of high-risk individuals is an important consideration for any organization involved in firesetters. To minimize the risk for future offending, there is a need for a collaborative, multiagency approach to the fire-setting behavior involving comprehensive risk assessment and appropriate referral for at-risk individuals [21]. In recent years, efforts have been made to develop effective treatment interventions aimed at replacing the fire-setting behavior with more socially acceptable ways of resolving problems [25]. To offer optimally targeted and effective treatment, the investigation of personality characteristics of the firesetter seems to be highly relevant. As far as the authors are aware, to date, only one study focusing on psychopathic traits of firesetters has been published. A study by Labree et al. [18] comprised 25 arsonists and 50 non-arsonists in a maximum security forensic hospital setting. The authors concluded that arsonists were more impulsive and showed less superficial charm and juvenile delinquency than non-arsonists.

The main aim of this study was to evaluate the prevalence of psychopathic traits in a consecutive sample of Finnish male pretrial fire-setting offenders. Our hypothesis was that, among firesetters, there would be a subgroup of individuals with high traits of psychopathy. Further, we hypothesized that fire-setting recidivists would show higher traits of psychopathy than one-time firesetters and that versatile firesetters would exhibit more features of psychopathy than exclusive firesetters.

## Methods

### Subjects and procedure

The forensic psychiatric examination reports of a consecutive sample of 135 male firesetters who underwent a pretrial forensic psychiatric evaluation during a 10-year period (1989 –1998) at Helsinki University Central Hospital were reviewed. Their primary offence concerned setting one or more fires, but the motive varied and hence we use the term “firesetter” instead of “arsonist” in this study. The Finnish forensic psychiatric examination report traditionally includes a paragraph summarizing the subject’s previous official criminal history. This official information was used in dividing the pretrial offenders into fire-setting recidivists and one-time firesetters and into exclusive and versatile firesetters. The firesetters were categorized as fire-setting recidivists if they had committed one or more separate fire-settings before the index one and as one-time firesetters if the index fire-setting was their first. Respectively, the firesetters were categorized as exclusive, if they had not been convicted of any other crimes and as versatile if they in addition to fire-settings also had other types of crime in their official criminal records.

### Psychopathic traits

Assessment of psychopathy-like personality character was performed using the 20-item Hare Psychopathy Checklist-Revised (PCL-R) [2], which has become the standard for assessing psychopathy in forensic settings. The PCL-R is a reliable and valid instrument for measuring psychopathy [26-29], and its psychometric properties appear to be much the same across countries [30]. Specific scoring criteria were used to rate each PCL-R item on a three-point scale (0 = absent, 1 = possibly or partially present, 2 = definitely present) according to the extent to which it applies to a given individual. The PCL-R items are summed to yield total scores ranging from zero to 40; scores of 30 and higher are considered diagnostic of psychopathy [31]. In line with recommendations of a lower cut-off score for European populations [32-36], a cut-off score of 25 has been used in studies performed in Scandinavian countries [36-38]. The PCL-R is underpinned by two factors that tap affective-interpersonal features (Factor 1 = The Affective-interpersonal factor: glibness and superficial charm, grandiose sense of self-worth, pathological lying, manipulative behavior, lack of remorse or guilt, shallow affect, lack of empathy, failure to accept responsibility) and socially deviant lifestyle and behaviors (Factor 2 = The Behavioral factor: proneness to boredom, parasitic lifestyle, poor behavioral controls, lack of realistic, long-term goals, impulsivity, irresponsibility, juvenile delinquency, revocation of conditional release). Although PCL-R assessments are recommended to be based on both a review of file information and a semi-structured interview with the offender, research has consistently shown that assessments based solely on file information are highly similar to those including an interview, and, provided that there is sufficient file information, are appropriate in the absence of an interview, especially for research purposes [26,39-41]. In this study, the forensic psychiatric examination reports were scored by one forensic psychiatrists of the research team (NL).

### Statistics

We conducted data analyses with the SPSS statistical software package version 19. We used the independent samples *t*-test, the Mann–Whitney test, the Likelihood ratio chi-square test (*χ*^2^), and Fisher’s exact test to compare the groups. The findings were considered significant when p < 0.05. The Bonferroni correction was not used to control Type I errors due to the multiple comparisons as it has been criticized for dramatically increasing the risk of Type II errors. Instead, effect sizes are reported. The phi (φ) coefficient was used as an effect size measure for the chi-square test and Fisher’s exact test and Cohen’s d for the independent samples *t*-test. For the Mann–Whitney *U*-test, the effect size measure used was theta (ϴ). The magnitude of the φ coefficient was interpreted as follows: 0.1 small effect, 0.3 moderate effect, and 0.5 large effect. Respectively, the magnitude of Cohen’s d was interpreted as follows: 0.2 small, 0.5, moderate; and 0.8 large effect [42-44]. ϴ can be interpreted as follows: 0.56 small, 0.64 moderate, and 0.70 large effect [45].

### Ethics

The study protocol was approved by the Ethics Committee of Helsinki University Hospital and pertinent institutional authorities.

## Results

The mean age of the firesetters was 32.3 years (SD 11.1, range 16–67). Forty-one firesetters (30.4%) were recidivists (mean number of separate fire-settings 3.6, SD 3.02, range 2–15). Fifty-four offenders (40.0%) were exclusive and 81 versatile firesetters. In Finland, the psychiatric classification according to the International Classification of Diseases- Ninth Revision (ICD-9) [46] was used in clinical practice between 1987 and 1995, after which it was replaced by ICD-10 [47]. According to the principal psychiatric diagnoses given in the forensic psychiatric examination, 6 offenders (4.4%) were mentally retarded (IQ ≤ 70), 30 (22.2%) were psychotic, 76 (56.3%) were personality-disordered and 22 (16.3%) suffered from some other psychiatric disorder (mood disorder, adjustment disorder, alcohol dependence, pyromania), and one (0.7%) had no psychiatric diagnoses. The persons with mental retardation were omitted from the PCL-R assessments. Thus, the final data comprised 129 male firesetters with normal IQ. Of these, 41 were fire-setting recidivists and 88 one-time firesetters, or 50 were exclusive and 79 versatile firesetters.

**Figure 1:**
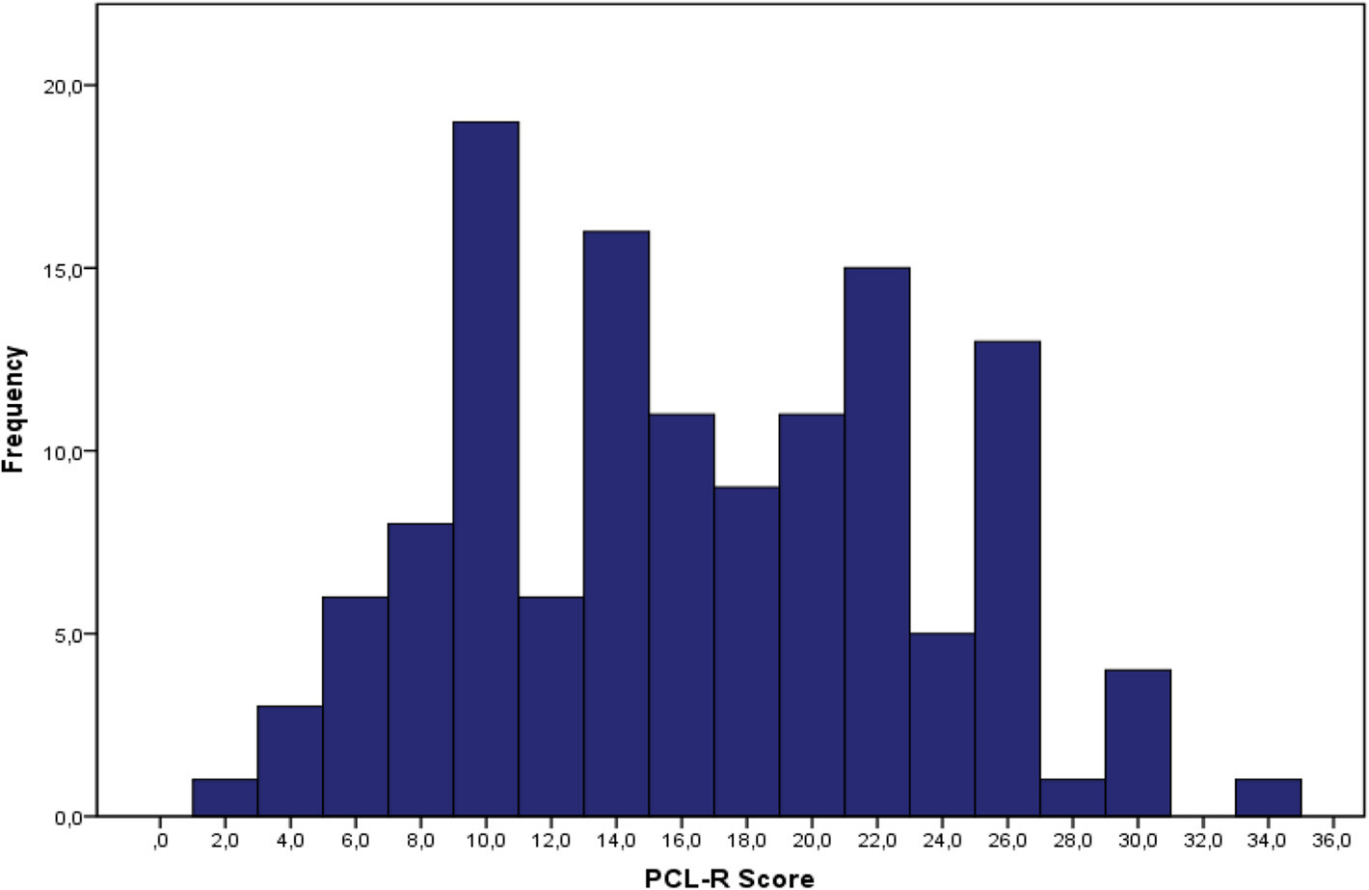
**Distribution of Hare Psychopathy Checklist-Revised (PCL-R) total scores in Finnish firesetters with normal IQ (n = 129).**

Of the 129 firesetters, 2 individuals (1.6%, 95% Cl: 0.0-3.7) scored ≥30 points, and 19 (14.7%, 95% Cl: 8.6-20.8) ≥ 25 points on the PCL-R. The mean PCL-R total score was 16.1 (SD 6.88, range 2–33), the mean Factor 1 score 5.0 (SD 3.41, range 0–13), and the mean Factor 2 score 9.9 (SD 3.86, range 1–18). The PCL-R distribution is presented in Figure 1.

In the subgroup of 88 one-time firesetters, 1 person (1.1%, 95% Cl: 0.0-3.4) scored ≥ 30 points and 11 (12.5%, 95% Cl: 5.6-19.4) ≥ 25 points. The mean PCL-R total score was 15.8 (SD 6.80, range 2–31), the mean Factor 1 score 5.1 (SD 3.59, range 0–13), and the mean Factor 2 score 9.7 (SD 3.89, range 1–18).

In the subgroup of 41 recidivists, 1 person (2.4%, 95%Cl: 0.0-7.2) scored ≥ 30 points and 8 (19.5%, 95% Cl: 7.4-31.6) ≥ 25 points. The mean PCL-R total score was 16.6 (SD 7.10, range 4–33), the mean Factor 1 score 4.8 (SD 3.01, range 0–12), and the mean Factor 2 score 10.3 (SD 3.80, range 0–18).

The comparisons between the recidivists and the one-time firesetters are presented in Table 1. No significant differences existed between the groups.

**Table 1:** **A comparison of one-time firesetters (n = 88) to fire-setting recidivists (n = 41) with normal IQ**

	**One-time firesetters**	**Fire-setting recidivists**	**Statistics** ^**1**^	**p**	**Effect size**
**Age** mean (SD)	32.0 (11.20)	33.5 (11.40)	t = 0.695	NS	d = −0.133
**Number of fire-settings** mean (SD)	1	3.5 (3.04)	t = −5.287	<0.001	d = −1.163
**Principal clinical diagnoses**					
Personality disorders	53/88	23/41	*χ* ^2^ = 0.063	NS	φ = 0.022 (max = 0.817)
Psychoses	20/88	10/41	*χ* ^2^ = 0.043	NS	φ = 0.018 (max = 0.376)
Other	15/88	8/41	*χ* ^2^ = 0.009	NS	φ = 0.008 (max = 0.318)
**PCL-R total score** mean (SD)	15.8 (6.8)	16.6 (7.1)	Z = −0.504	NS	ϴ = 0.472
**PCL-R total score ≥ 30**	1/88	1/41	#	NS	NA
**PCL-R total score ≥ 25**	11/88	8/41	*χ* ^2^ = 1.095	NS	φ = 0.092 (max = 0.284)
**PCL-R factor 1** mean (SD)	5.1 (3.59)	4.8 (3.01)	Z = −0.089	NS	ϴ = 0.495
**PCL-R factor 2** mean (SD)	9.7 (3.89)	10.3 (3.80)	Z = −0.840	NS	ϴ = 0.454
**Item** mean (SD)					
**1. Glibness/superficial charm**	0.23 (0.45)	0.29 (0.60)	Z = −0.215	NS	ϴ = 0.492
**2. Grandiose sense of self worth**	0.45 (0.61)	0.29 (0.56)	Z = −1.619	NS	ϴ = 0.426
**3. Need for stimulation**	1.16 (0.79)	1.17 (0.77)	Z = −0.038	NS	ϴ = 0.498
**4. Pathological lying**	0.11 (0.41)	0.07 (0.35)	Z = −0.630	NS	ϴ = 0.485
**5. Conning/manipulative**	0.51 (0.82)	0.29 80.68)	Z = −1.581	NS	ϴ = 0.433
**6. Lack of remorse or guilt**	0.92 (0.75)	1.06 (0.84)	Z = −0.878	NS	ϴ = 0.412
**7. Shallow affect**	0.72 (1.73)	0.93 (0.82)	Z = −1.369	NS	ϴ = 0.430
**8. Callous/ lack of empathy**	0.80 (0.77)	0.84 (0.72)	Z = −0.368	NS	ϴ = 0.481
**9. Parasitic lifestyle**	0.75 (0.79)	0.71 (0.68)	Z = −0.276	NS	ϴ = 0.486
**10. Poor behavioral controls**	1.59 (0.66)	1.66 (0.58)	Z = −0.411	NS	ϴ = 0.482
**11. Promiscuous sexual behavior**	0.28 (0.56)	0.36 (0.74)	Z = −0.107	NS	ϴ = 0.496
**12. Early behavior problems**	0.80 (0.89)	0.98 (0.88)	Z = −1.071	NS	ϴ = 0.469
**13. Lack of realistic goals**	1.24 (0.76)	1.39 (0.77)	Z = −1.159	NS	ϴ = 0.442
**14. Impulsivity**	1.65 (0.55)	1.76 (0.49)	Z = −1.140	NS	ϴ = 0.451
**15. Irresponsibility**	1.75 (0.49)	1.61 (0.63)	Z = −1.191	NS	ϴ = 0.450
**16. Failure to accept responsibility**	1.24 (0.70)	1.20 (0.68)	Z = −0.425	NS	ϴ = 0.479
**17. Many short-term marital relationships**	0.07 (0.33)	0.10 (0.30)	Z = −1.089	NS	ϴ = 0.475
**18. Juvenile delinquency**	0.48 (0.84)	0.57 (0.90)	Z = −0.596	NS	ϴ = 0.471
**19. Revocation of conditional release**	0.28 (0.69)	0.34 (0.76)	Z = −0.363	NS	ϴ = 0.488
**20. Criminal versatility**	1.00 (0.94)	1.02 (0.99)	Z = −0.140	NS	ϴ = 0.493

^1^The independent samples *t*-test (t), Mann–Whitney *U*-test (Z), Fisher’s exact test (#) and chi square-test (*χ* ^2^) are used for comparing the groups. NS = not statistically significant. NA = not applicable. Effect sizes are reported, d = Cohen’s d, φ = phi and its maximum value, ϴ = theta.

Age, number of fire-settings, principal diagnostic groups, and PCL-R total, factor, and item-by-item scores among one-time firesetters and recidivists are presented.

Among the 50 exclusive firesetters, no one (0.0%) scored ≥ 30 points, but 1 person (2.0%, 95% Cl: 0.0-5.9) scored ≥ 25 points. The mean PCL-R total score was 12.4 (SD 5.85, range 2–29), the mean Factor 1 score 3.7 (SD 2.99, range 0–12), and the mean Factor 2 score 8.3 (SD 3.36, range 1–15).

Among the 79 versatile firesetters, 2 persons (2.5%, 95% Cl: 0.0-6.0) scored ≥ 30 points, and 18 scored (22.8%, 95% Cl: 13.5-32.0) ≥ 25 points. The mean PCL-R total score was 18.4 (SD 6.51, range 5–33), the mean Factor 1 score 5.8 (SD 3.44, range 0–13), and the mean Factor 2 score 10.8 (SD 3.86, range 4–18).

The comparisons between persons who had been convicted for fire-settings only and those with criminal versatility are presented in Table 2. The firesetters with criminal versatility exhibited significantly higher PCL-R total scores and factor scores. Also, the prevalence of persons scoring ≥25 points on the PCL-R was greater among versatile firesetters. Focusing on specific items, firesetters with criminal versatility exhibited significantly higher levels of glibness/superficial charm, need for stimulation, lack of remorse or guilt, lack of empathy, poor behavioral controls, impulsivity, irresponsibility, failure to accept responsibility, and juvenile delinquency.

**Table 2:** **A comparison of exclusive firesetters (n = 50) to those with criminal versatility (n = 79)**

	**Exclusive firesetters**	**Firesetters with criminal versatility**	**Statistics** ^**1**^	**p**	**Effect size**
**Age** mean (SD)	31.9 (9.26)	32.9 (12.33)	t = 0.493	NS	d = −0.092
**Number of fire-settings** mean (SD)	2.2 (2.88)	1.5 (1.26)	t = −1.671	NS	d = 0.315
**Principal clinical diagnoses**					
Personality disorders	23/50	53/79	*χ* ^2^ = 5.626	0.03	φ = 0.209 (max = 1.050)
Psychoses	14/50	16/79	*χ* ^2^ = 1.030	NS	φ = 0.089 max = 0.692)
Other	13/50	10/79	*χ* ^2^ = 3.720	NS	φ = 0.170 (max = 0.586)
**PCL-R total score** mean (SD)	12.4 (5.83)	18.4 (6.52)	Z = −4.752	<0.001	ϴ = 0.252
**PCL-R total score ≥ 25**	1/50	18/79	#	<0.001	NA
**PCL-R factor 1** mean (SD)	3.7 (2.99)	5.8 (3.44)	Z = −3.435	<0.001	ϴ = 0.321
**PCL-R factor 2** mean (SD)	8.3 (3.36)	10.8 (3.86)	Z = −3.378	<0.001	ϴ = 0.324
**Item** mean (SD)					
**1. Glibness/superficial charm**	0.1 (0.44)	0.3 (0.52)	Z = −2.847	0.004	ϴ = 0.393
**2. Grandiose sense of self worth**	0.4 (0.64)	0.4 (0.57)	Z = −0.694	NS	ϴ = 0.470
**3. Need for stimulation**	1.0 (0.79)	1.3 (0.75)	Z = −2.330	0.02	ϴ = 0.385
**4. Pathological lying**	0.1 (0.42)	0.1 (0.38)	Z = −0.312	NS	ϴ = 0.493
**5. Conning/manipulative**	0.4 (0.72)	0.5 (0.82)	Z = −0.928	NS	ϴ = 0.463
**6. Lack of remorse or guilt**	0.6 (0.72)	1.2 (0.74)	Z = −3.908	<0.001	ϴ = 0.299
**7. Shallow affect**	0.7 (0.80)	0.8 (0.74)	Z = −0.642	NS	ϴ = 0.469
**8. Callous/ lack of empathy**	0.6 (0.65)	1.0 (0.77)	Z = −2.717	0.007	ϴ = 0.364
**9. Parasitic lifestyle**	0.9 (1.37)	0.8 (0.78)	Z = −0.248	NS	ϴ = 0.488
**10. Poor behavioral controls**	1.5 (0.68)	1.7 (0.58)	Z = −2.436	0.02	ϴ = 0.396
**11. Promiscuous sexual behavior**	0.2 (0.47)	0.4 (0.69)	Z = 2.024	0.04	ϴ = 0.423
**12. Early behavior problems**	0.8 (0.85)	0.9 (0.92)	Z = −0.313	NS	ϴ = 0.485
**13. Lack of realistic goals**	1.1 (0.81)	1.4 (0.72)	Z = −1.664	NS	ϴ = 0.420
**14. Impulsivity**	1.4 (0.64)	1.9 (0.36)	Z = −4.366	<0.001	ϴ = 0.320
**15. Irresponsibility**	1.5 (0.68)	1.9 (0.35)	Z = −3.953	<0.001	ϴ = 0.343
**16. Failure to accept responsibility**	1.0 (0.71)	1.4 (0.63)	Z = −3.345	0.001	ϴ = 0.338
**17. Many short-term marital relationships**	0.1 (0.31)	0.1 (0.33)	Z = −0.798	NS	ϴ = 0.483
**18. Juvenile delinquency**	0.3 (0.71)	0.6 (0.93)	Z = −2.181	0.03	ϴ = 0.412
**19. Revocation of conditional release**	0.1 (0.50)	0.4 (0.81)	Z = −1.926	NS	ϴ = 0.437
**20. Criminal versatility**	0.3 (0.71)	1.5 (0.80)	Z = −6.781	<0.001	ϴ = 0.180

^1^The independent samples *t*-test (t), Mann–Whitney *U*-test (Z), Fisher’s exact test (#) and chi square-test (*χ* ^2^) are used for comparing the groups. NS = not statistically significant. NA = not applicable. Effect sizes are reported, d = Cohen’s d, φ = phi and its maximum value, ϴ = theta.

Age, number of fire-settings, principal diagnostic groups, and PCL-R total, factor, and item-by-item scores among exclusive and versatile firesetters are presented.

## Discussion

### Prevalence and level of psychopathy

Our first hypothesis was that we would find a subgroup of firesetters who exhibit high traits of psychopathy. Based on the recommendation of a lower cut-off score for European populations, approximately 15% of the study sample exhibited significant traits of psychopathy. However, only 1.6% of the firesetters scored 30 points or more, which is the original cut-off score of psychopathy by Hare [31]. The proportion of these highly psychopathic persons seems to be smaller in the present sample than in a Finnish prison population in general (12.3%) [48].

The mean PCL-R total score of the sample was 16.1 (SD 6.88). It is, of course, difficult to compare the scorings from other studies to ours, but in a Dutch study by Labree et al. [18], the mean PCL-R total score for arsonists was slightly higher (mean 17.4) than that observed in our sample. The sample of Labree et al. comprised firesetters sentenced to forced treatment in a maximum security forensic hospital with higher prevalence rates of both personality and psychotic disorders than observed in the present sample. The authors found that the level of psychopathic traits did not significantly differ between the patients with arsons and those with other criminal acts. In our sample, the level of psychopathic traits seemed to be slightly lower than reported previously among Finnish prisoners (mean 19.5, SD 8.05) [48].

Factor 1, which measures affective and interpersonal features of psychopathy, is often regarded as “true” or “core” psychopathy. Indeed, glibness, superficial charm, grandiose sense of self-worth, pathological lying, manipulative behavior, lack of remorse or guilt, shallow affect, lack of empathy, and failure to accept responsibility describe well the original prototype of a psychopathic person provided by Cleckley [1]. Factor 2 describes a socially deviant lifestyle and behaviors and, in many ways, reflects antisocial behavior. In the present study, the mean PCL-R Factor 1 score was 5.0 (SD 3.41) and Factor 2 score 9.9 (SD 3.86). Among the firesetters in the study by Labree et al. [18], the mean factor scores were 7.8 and 8.4, respectively, and no statistical difference was observed between the arsonists and non-arsonists. In a Finnish prison study [48], the corresponding mean factor scores were 7.1 (SD 3.70) and 9.7 (SD 4.8). This implies that as a group pretrial firesetters exhibit less affective and interpersonal features of psychopathy, but nearly equal amounts of antisocial behavior as a prison population in general.

### Psychopathy and fire-setting recidivism

Approximately 30% of the sample was fire-setting recidivists. The point prevalence of psychopathy as well as the level of psychopathic traits did not significantly differ between the fire-setting recidivists and those with only one fire-setting. Furthermore, the affective-interpersonal features of psychopathy or the amount of antisocial behavior did not significantly differ between these two groups. So, our hypothesis that fire-setting recidivists would show higher traits of psychopathy than one-time firesetters was not supported. Interestingly, focusing on single items of the PCL-R, the fire-setting recidivists did not significantly differ from the one-time firesetters on impulsivity (PCL-R item 14) nor need for stimulation (item 3).

### Psychopathy and versatile firesetters

Our third hypothesis was that versatile firesetters would exhibit higher traits of psychopathy than those with criminal exclusivity. Indeed, with a cut-off score of 25, the firesetters with criminal versatility exhibited a significantly higher point prevalence of psychopathy than did those with criminal exclusivity. The versatile firesetters also exhibited significantly more affective-interpersonal features of psychopathic character as well as antisocial behavior than the exclusive ones. One must remember, that versatility itself is one of the 20 PCL-R items, thus affecting the total PCL-R score, but this item loads to neither factors. Our finding is much in line with earlier psychopathy research reporting that offenders with psychopathic character show a greater variety of crimes than those exhibiting low traits of psychopathy [49,50]. In a recent study by Ducat et al. [22], versatile firesetters were more often diagnosed with personality disorders than exclusive ones, which was also observed in the present study. Ducat et al. concluded that versatile firesetters may have a more chronic course of antisocial behavior, which is much in line with our finding.

According to the study by Labree et al. [18], arsonists were significantly more prone to impulsive behavior than non-arsonists. Furthermore, they showed less superficial charm and juvenile delinquency than non-arsonists. In our sample, prone impulsivity was observed among versatile firesetters when comparing them to exclusive ones. On the other hand, exclusive firesetters exhibited significantly less superficial charm and juvenile delinquency than those with criminal versatility.

In the Multi-Trajectory Theory of Adult Firesetting (M-TTAF) by Gannon et al. [51], they summarized five associated prototypical fire-setting trajectories: antisocial cognition, grievance, fire interest, emotionally expressive/need for recognition and a multifaceted type of trajectory. M-TTAF describes four key psychological issues likely to be involved with fire-setting: inappropriate fire interests/scripts, offense-supportive cognition, self/emotional regulation issues and communication problems. Offense-supportive attitudes and values are considered prominent risk factors for fire-setting behavior and might clinically be expressed as antisocial personality or conduct disorders or as antisocial attitudes or values [51]. In our study, a subgroup expressing high PCL-R ratings was identified and it might be that their fire-setting behavior evolved out of an antisocial cognition or multifaceted trajectory rather than from fire interest. This was also supported by the fact that they were mainly versatile firesetters, expressing other forms of criminal behavior as well, in addition to fire-setting. These are motivational factors that need to be addressed when planning treatment for firesetters expressing different levels of psychopathy.

### Limitations

In Finland, approximately 500–600 arson attempts are made each year, and 100 offenders are convicted of the crime. The proportion of individuals undergoing a forensic psychiatric evaluation of all firesetters suspected by the police has been estimated to be only 10% [52]. Hence, the present sample is not representative of firesetters in general.

The sample was drawn from a single hospital and all pretrial offenders were men. Only the principal psychiatric diagnoses were collected from the forensic psychiatric reports but many pretrial offenders showed psychiatric comorbidity. That is, a person might primarily exhibit a psychotic disorder and, in addition to this, a comorbid personality disorder. However, the main focus of the present study was on the frequency and level of psychopathy among firesetters, not the relationship between psychopathy and the ICD-diagnostics. However, offenders with mental retardation were omitted from the study since it is questionable if a person with abnormally low IQ can be scored with the PCL-R.

The Finnish forensic psychiatric examination statement traditionally includes a paragraph summarizing the subject’s previous official criminal history. This official information was used in dividing the pretrial offenders into fire-setting recidivists and one-time firesetters and into exclusive and versatile firesetters. During the pretrial psychiatric examination, some firesetters described crimes that were not recorded in their official criminal history. These crimes were taken into account in the PCL-R ratings, which explains that the mean score in the “versatility” item among exclusive firesetters was not zero, but 0.3 (vs. 1.5 in versatile firesetters). However, because the authors were not able to clarify, if these “confessions” were true or not, splitting the offenders into different subgroups was done with the information gathered from official documents.

The sample included two persons under 18 years of age. They were also assessed using the PCL-R instead of the Psychopathy Check-List- Youth Version (PCL-YV) [53]. No inter-rater reliability was calculated as the PCL-R ratings were carried out by one experienced forensic psychiatrist trained for these assessments. One must also remember that the study was cross-sectional. The firesetters were categorized as fire-setting recidivists if they had committed one or more separate fire-settings before the index one and as one-time firesetters if the index fire-setting was their first one. It is possible that some of the first-timers set fires later in life, and by doing so, became recidivists. Accordingly, the men were categorized as exclusive or versatile firesetters. Some exclusive firesetters may have been convicted of other types of crime later in life, thus becoming versatile firesetters. To shed light on these issues, a prospective follow-up study design should be chosen in the future.

## Conclusions

In our study, we found that among firesetters, there is a subgroup of persons with significant psychopathic traits, which should be recognized in legal and health care organizations. These firesetters are likely to be motivated by antisocial pathways rather than by fire interest. Although psychopathy was associated with greater criminal versatility, it bore no relationship to fire-setting recidivism.
